# Hæmorrhagic Diathesis

**Published:** 1852-12

**Authors:** N. S. Davis

**Affiliations:** Chicago


*Hæmorrhagic Diathesis.*

Messrs. Editors:—In the July number of your Journal I reported (see page 110) a case of spontaneous hæmorrhage into the tissues, with a brief account of the symptoms and history of the patient. I then promised to inform you further in regard to the treatment of the case at some future time. From previous observation of cases of this diathesis, I had been led to regard the essential fault as consisting more in a want of contractility in the capillary blood-vessels, than in the constitution of the blood. In the case to which I allude, the oft-repeated bleedings from the nose and elsewhere had induced a well marked anemic condition of the blood, which now co-operated with the want of contractility of the capillaries in increasing the hæmorrhagic condition. Hence we had two indications to fulfil by treatment, viz.: to improve the quality of the blood, and to increase the tonicity and contractility of the capillaries. To accomplish the first, I directed him a plain nutritious diet consisting of animal broth well salted, and milk, with the Tinct. Ferri Murias. To accomplish the second object, I directed the Tincture of Ergot, which was suggested by the alleged fact, that Ergot when eaten with bread, &c., induces *dry gangrene* and other bad consequences by its power to contract the fibrous tissues, and thereby lessen the capillary circulation especially in the extremities. Acting on this thought, I prescribed for the patient a mixture of equal parts of Tinct. Ferri Murias, and Tinct. Ergot, 30 drops to be taken three times a day. The bloody tumor on the shoulder and back, I directed to be bathed daily in a simple solution of common salt in water. This treatment was continued steadily for five or six weeks, and the patient rapidly improved in health and strength. The tumor gradually diminished in size, and at the end of three weeks from my first visit he was able to resume his usual employment. During the next week after he began to work, and while he was still under treatment, he bruised one leg so as to lacerate the parts slightly. It was more disposed to bleed than in healthy persons, but one or two applications of a cold solution of Tannin promptly arrested the oozing of blood and the parts healed. With this exception, no hæmorrhage either from the nose or elsewhere occurred during the six weeks he was under treatment. At the end of that time I lost sight of the patient until a day or two since. On inquiring, I learned that he had been able to continue his labor in a moderate degree until the present time, and had taken no medicine since the time above indicated. During the last two months his disposition to hæmorrhage has again increased, indicated by a return of epistaxis every few days. He is not now under treatment, but I have made arrangements to procure some of his blood for minute examination, so soon as a spontaneous hæmorrhage occurs of sufficient quantity. So you may hear from the case again.

Yours truly,

Chicago, Dec. 10, 1852. N. S. DAVIS, M.

